# Advanced biological age is associated with improved antibody responses in older high-dose influenza vaccine recipients over four consecutive seasons

**DOI:** 10.1186/s12979-022-00296-7

**Published:** 2022-08-23

**Authors:** Chris P. Verschoor, Daniel W. Belsky, Melissa K. Andrew, Laura Haynes, Mark Loeb, Graham Pawelec, Janet E. McElhaney, George A. Kuchel

**Affiliations:** 1 Health Sciences North Research Institute, 41 Ramsey Lake Road, Rm 32033, Sudbury, ON P3E 5J1 Canada; 2 Northern Ontario School of Medicine, Sudbury, ON Canada; 3 Department of Epidemiology, Columbia University Mailman School of Public Health, New York, NY USA; 4 Robert N. Butler Columbia Aging Center, Columbia University, New York, NY USA; 5 Department of Medicine, Dalhousie University, Halifax, NS Canada; 6 UConn Center on Aging, University of Connecticut School of Medicine, Farmington, CT USA; 7 Department of Pathology and Molecular Medicine, McMaster University, Hamilton, ON Canada; 8 Department of Immunology, University of Tübingen, Tübingen, Germany

**Keywords:** Biological age, Influenza, Vaccination, Older adults, Cytomegalovirus, Canadian longitudinal study on aging

## Abstract

**Background:**

Biological aging represents a loss of integrity and functionality of physiological systems over time. While associated with an enhanced risk of adverse outcomes such as hospitalization, disability and death following infection, its role in perceived age-related declines in vaccine responses has yet to be fully elucidated. Using data and biosamples from a 4-year clinical trial comparing immune responses of standard- and high-dose influenza vaccination, we quantified biological age (BA) prior to vaccination in adults over 65 years old (*n* = 292) using a panel of ten serological biomarkers (albumin, alanine aminotransferase, creatinine, ferritin, free thyroxine, cholesterol, high-density lipoprotein, triglycerides, tumour necrosis factor, interleukin-6) as implemented in the BioAge R package. Hemagglutination inhibition antibody titres against influenza A/H1N1, A/H3N2 and B were quantified prior to vaccination and 4-, 10- and 20- weeks post-vaccination.

**Results:**

Counter to our hypothesis, advanced BA was associated with improved post-vaccination antibody titres against the different viral types and subtypes. However, this was dependent on both vaccine dose and CMV serostatus, as associations were only apparent for high-dose recipients (d = 0.16–0.26), and were largely diminished for CMV positive high-dose recipients.

**Conclusions:**

These findings emphasize two important points: first, the loss of physiological integrity related to biological aging may not be a ubiquitous driver of immune decline in older adults; and second, latent factors such as CMV infection (prevalent in up to 90% of older adults worldwide) may contribute to the heterogeneity in vaccine responses of older adults more than previously thought.

**Supplementary Information:**

The online version contains supplementary material available at 10.1186/s12979-022-00296-7.

## Background

Lower respiratory tract infections are one of the leading causes of total disease burden in the world, especially so for older adults [1]. Prior to the COVID-19 pandemic, influenza accounted for more than 70% of all disability-adjusted life years associated with communicable diseases in European adults over 65 [2], and between 2010 and 2020, influenza caused approximately 4 million hospitalizations and 340,000 deaths in the United States alone [3]. Seasonal vaccination remains our best preventative measure against influenza, although there is significant heterogeneity in its effectiveness. For example, while protection against influenza-like illness is mostly similar across ages for all viral subtypes [4], vaccination is less effective against hospitalization for older adults [5], especially so for A/H3N2 and when there is a mismatch with the circulating strain [6]. Underlying health conditions can also contribute to lower vaccine effectiveness in older adults, evident by reduced protection against hospitalization with increasing frailty [7]. It is not clear why vaccination becomes less efficacious with age, although impairments in the ability to induce strong antibody-responses to the vaccine are likely a major contributor [8]. Nonetheless, growing evidence indicates that immune deficits most greatly contributing to an increased risk of hospitalization, disability and death involve cell- as opposed to antibody-mediated systems, the latter of which is typically evaluated as an easily measurable predictor of sterilizing immunity [9].

Given the complexity of the immune system, it is not surprising that the manner in which immunity changes with age is also complex and multifactorial. For example, innate immune cells such as monocytes tend to exhibit a hyperinflammatory phenotype with reduced signalling capacity [10], while the proliferative capacity of T- and B-lymphocytes decline [11]. The architecture of primary and secondary lymphoid organs are also broadly affected, compromising the efficiency of intracellular communication and the magnitude and balance of cellular output [12]. Modern theories in aging research suggest that a single fundamental mechanism underlying these alterations is unlikely and, instead, propose a network of interrelated age-related biological phenomena [13–15]. Consistent with this theoretical perspective, several hallmarks of aging [14] are related to impaired immune function, including disruptions to the proteostasis network impacting efficient antigen presentation [16], dysregulated metabolism on T/B-cell function [17, 18], and reduced telomere length and the decline in T-cell clonal expansion [19]. Further, proxy measures of biological aging, which represents the overall loss of physiological integrity due to the accumulation of these hallmarks, have been shown to be positively correlated with the likelihood of SARS-CoV-2 infection [20] and the severity of disease [20–22].

We hypothesized that biological aging is an important determinant of vaccine effectiveness in older adults, and specifically, vaccine immunogenicity will be most impaired in those with the most advanced biological age. To test this, we employed data and biosamples from our 4-year clinical trial comparing standard- to high-dose influenza vaccine in adults 65 and older and estimated the association between biological age and antibody titres pre- and post-vaccination. Given that CMV infection is often correlated with weaker influenza vaccine responses [23], we also investigated a potential interaction between CMV and biological age in associations with vaccine immunogenicity.

## Results

### Participant characteristics and blood biomarker measures

This study was a nested analysis of a 4-year randomized vaccine trial, in which 246 unique individuals enrolled, many over multiple years. Thus, the total number of participant enrollments over the course of the trial was 612. From this, we randomly selected 300 participant enrollments, which represents 166 unique individuals. Of those 166, 5 participated in 4 years, 32 in 3 years, 55 in 2 years and 74 only in a single year. Of the 300 participants included in the current study, complete data for blood biomarkers was available for *n* = 292, which formed our analytical sample (mean age = 76, 65% women, mean BMI = 28, 56% CMV positive; Table 1). The average frailty index was 0.11, which falls on the classification threshold for robust or pre-frail [24]. Participant characteristics in the analysis sample were similar to those in the parent trial (*p* > 0.05 for all comparisons), and laboratory-confirmed influenza was identified in 16 (5.5%), 11 of whom were identified as carrying A/H1N1 or A/H3N2, and 5 of whom were identified as carrying B.

**Table 1:** Characteristics of the participant enrollments in the current study and that of the parent trial

	Current subset (***N*** = 292)	Parent trial (***N*** = 612)
**Age**	76 (7.11)	76 (7.4)
**Sex**		
Female	190 (65.1%)	410 (67.0%)
Male	102 (34.9%)	202 (33.0%)
**BMI (kg/m**^**2**^**)**	28 (4.72)	28 (4.87)
Missing	1 (0.3%)	3 (0.5%)
**CMV serostatus**		
Negative	130 (44.5%)	287 (46.9%)
Positive	162 (55.5%)	325 (53.1%)
**Frailty Index**	0.11 (0.0708)	0.11 (0.0734)
Missing	0 (0%)	2 (0.3%)
**Dose**		
Standard	145 (49.7%)	316 (51.6%)
High	147 (50.3%)	296 (48.4%)
**Site**		
HSNRI	184 (63.0%)	356 (58.2%)
UCHC	108 (37.0%)	256 (41.8%)
**Year**		
2014/2015	58 (19.9%)	106 (17.3%)
2015/2016	79 (27.1%)	175 (28.6%)
2016/2017	83 (28.4%)	174 (28.4%)
2017/2018	72 (24.7%)	157 (25.7%)

Continuous data is summarized as the mean (standard deviation), whereas categorical data is the count (frequency)

For each blood biomarker, geometric means, standard deviations, and their correlation with chronological age in the vaccine-trial sample and the Canadian Longitudinal Study on Aging (CLSA) training sample (described in the methods) are shown in Table 2. Analyte concentrations and correlations with chronological age were similar between cohorts, with some exceptions. For example, concentration of ALT and FERR were approximately 30–50% lower in the vaccine cohort as compared to the CLSA, while TNF was approximately 10 times higher. Given the large discrepancy for TNF and differences in assay methodology, values were re-scaled so that the average concentration and standard deviation were matched to that of the CLSA. We performed the same procedure with values of IL-6. Correlations with chronological age were similar between cohorts, with the exception of FERR and TRIG, both of which correlated more strongly in the vaccine cohort.

**Table 2:** Summary of biomarker measures used to estimate biological aging

	Vaccine cohort		CLSA	
	Female	Male	Female	Male
	(***N*** = 190)	(***N*** = 102)	(***N*** = 1928)	(***N*** = 1938)
**Albumin (ALB), g/L**	42.5 ± 1.09 [-0.25**]	42.8 ± 1.07 [- 0.4***]	39.6 ± 1.07 [- 0.14***]	39.7 ± 1.07 [- 0.15***]
**Alanine aminotransferase (ALT), U/L**	13.2 ± 1.60 [- 0.26**]	15.0 ± 1.51 [- 0.45***]	18.5 ± 1.45 [- 0.13***]	21.4 ± 1.46 [- 0.23***]
**Creatinine (CREAT),** **µmol/L**	72.0 ± 1.27 [0.15]	87.4 ± 1.32 [0.02]	72.0 ± 1.21 [0.15***]	91.2 ± 1.23 [0.16***]
**Ferritin (FERR), µg/L**	52.4 ± 2.19 [- 0.2*]	78.7 ± 2.43 [- 0.22]	104.5 ± 2.17 [- 0.04*]	159.2 ± 2.22 [- 0.04]
**Thyroxine (T4), pmol/L**	16.4 ± 1.24 [- 0.02]	15.7 ± 1.28 [0.26*]	15.4 ± 1.19 [0.06**]	14.9 ± 1.16 [0.04]
**Cholesterol (CHOL), mmol/L**	4.7 ± 1.25 [- 0.13]	4.2 ± 1.26 [- 0.29*]	5.3 ± 1.24 [- 0.09***]	4.5 ± 1.27 [- 0.11***]
**High-density lipoprotein (HDL), mmol/L**	1.4 ± 1.30 [0.07]	1.2 ± 1.31 [0.23]	1.6 ± 1.33 [0.02]	1.3 ± 1.34 [0.06*]
**Triglycerides (TRIG), mmol/L**	1.6 ± 1.51 [- 0.22*]	1.8 ± 1.71 [- 0.51***]	1.5 ± 1.58 [- 0.03]	1.5 ± 1.65 [- 0.11***]
**Tumour necrosis factor (TNF)**^**a**^**, pg/mL**	11.2 ± 1.37 [0.19*]	12.1 ± 1.42 [- 0.03]	1.1 ± 1.43 [0.21***]	1.1 ± 1.39 [0.23***]
**Interleukin 6 (IL-6)**^**a**^**, pg/mL**	3.0 ± 2.79 [0.14]	2.9 ± 1.99 [0.05]	2.3 ± 1.78 [0.16***]	2.3 ± 1.77 [0.24***]

The geometric mean ± standard deviation is presented for each biomarker measure. The correlation with chronological age (shown in square brackets) represents the standardized coefficient for age from a fixed (ie. CLSA) or mixed effect model including the random intercept for participant (ie. vaccine cohort); significance as follows: ***, *p* < 0.001; **, *p* < 0.01; *, p < 0.05

^a^represents the concentrations prior to rescaling

### Biological age and its association with participant demographics

**Fig. 1:**
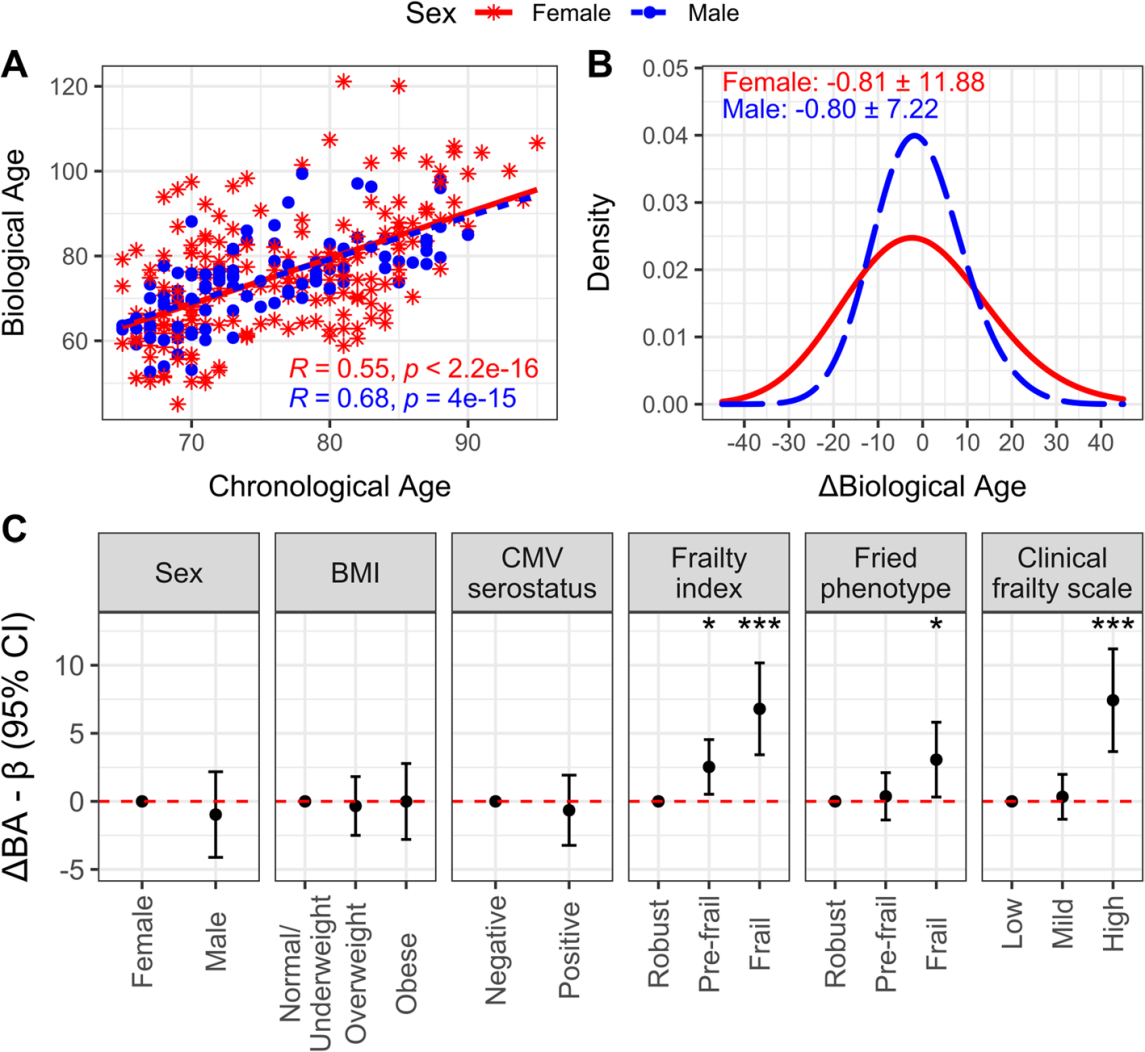
Estimation of biological aging in older vaccine recipients. For females (red, asterisks, solid line) and males (blue, circles, dashed line), **A** Pearson’s correlation (R) of chronological age with biological age (BA) is presented, along with **B**) the mean and standard deviation of the difference in BA from chronological age (ie. ΔBA). **C** The difference in BA and 95% confidence interval for categories of health-related factors relative to their reference (ie. the first category shown), was estimated using mixed model regression including a random intercept for participant. ***, *p* < 0.001; **, *p* < 0.01; *, *p* < 0.05

Vaccine trial participants’ biological age were correlated with their chronological ages in both women (Pearson’s *r* = 0.55, *p* < 0.001) and men (*r* = 0.68, *p* < 0.001) (Fig. 1A), and the average difference in biological age and chronological age (ΔBA) was - 0.8 ± 11.9 years (min/max = - 24/40) for women, and - 0.8 ± 7.2 years (- 17/21) for men (Fig. 1B). Although biological age was not associated with participant sex, BMI, or CMV serostatus, participants who were biologically older were more likely to be pre-frail or frail; for example, participants classified as frail (or high frailty) according to the frailty index, Fried’s frailty phenotype or Clinical Frailty Scale were 3.1 to 7.4 years older than those classified as robust (or low frailty) by those respective measures (Fig. 1C).

We also investigated whether biological age was associated with the incidence of laboratory-confirmed influenza 10- to 20-weeks post-vaccination. Only minor, non-significant associations were observed when comparing the mean ΔBA between participants that tested positive for any virus (- 0.56 ± 11.2, *n* = 16), influenza A only (0.31 ± 11.2, *n* = 11), or flu B only (- 2.5 ± 12.1, *n* = 5), with those that did not test positive (- 0.82 ± 10.5, *n* = 284).

### Associations between biological age and influenza vaccine responses

**Fig. 2:**
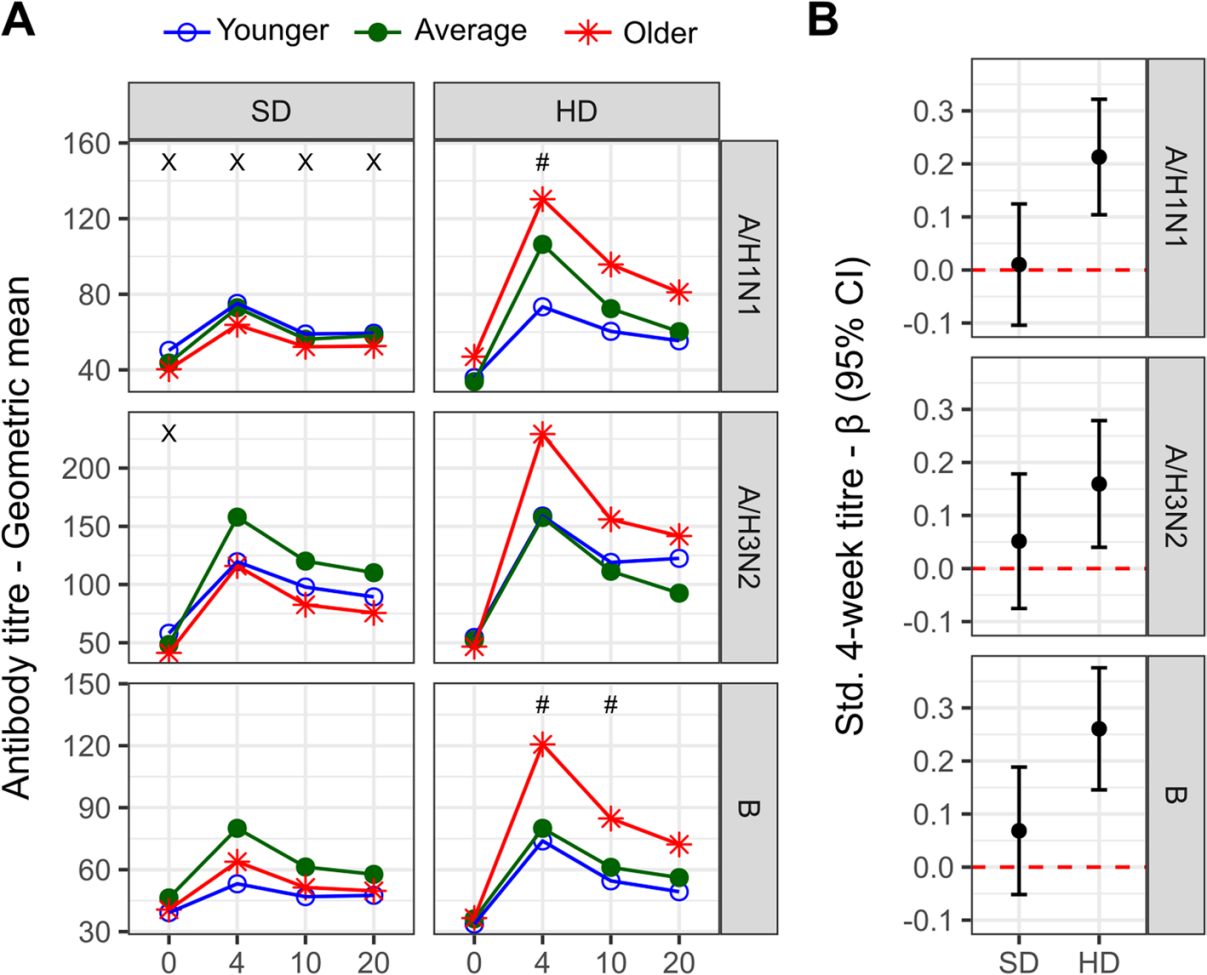
Vaccine antibody responses against A/H1N1, A/H3N2 and B are associated with biological aging in high-dose recipients. **A** The geometric mean titres in standard-dose (SD) and high-dose (HD) recipients pre-vaccination (ie. 0) and 4-, 10- and 20-weeks post-vaccination in older adults stratified by degree of biological aging; significance of trend test indicated by X (negative association) or # (positive association). **B** Standardized coefficients and 95% confidence intervals for the association between the natural-log 4-week titre and BA, adjusted for the fixed effect of baseline titre and other covariates; lack of overlap with the red dotted line indicates significance

To investigate the association of pre-vaccination biological age with vaccine responses at 4-, 10- and 20-weeks post-vaccination, we first assessed the trends in antibody-titres between participant tertiles, which we categorized as younger (ΔBA range: women = - 23.9 to - 5.9, men = - 16.8 to - 4.6), average (women = - 5.7 to 3.3, men = - 4.6 to 1.9) or older (women = 3.3 to 40.1, men = 1.93 to 21.3) biological age (Fig. 2A). For standard-dose recipients, there was no obvious trend in differences between pre- or post-vaccination titres of participants classified as younger, average or older biological age, with the exception of titres against A/H1N1, which were higher in younger biological aged participants at all time points (*p* < 0.05). For high-dose recipients, those with older biological ages tended to exhibit higher antibody titres post-vaccination, and a trend of increasing titres with biological age (ie. younger > average > older) was significant at 4-weeks for A/H1N1 and 4- and 10-weeks for B (*p* < 0.05).

We next used mixed-effects regression to test if participants’ biological age were related to changes in antibody titres from baseline to 4-weeks post-vaccination, adjusting for age and sex (Fig. 2B). Although no associations were apparent for standard-dose recipients, for those receiving high-dose vaccination older biological age was associated with increased antibody titers from baseline to follow-up for all viral subtypes; for every 1-standard deviation greater ΔBA, the change in natural-log antibody titres against A/H1N1, A/H3N2, and B increased by 0.21 (95%CI: 0.10, 0.32), 0.16 (0.04, 0.28), and 0.26 (0.15, 0.38) standard deviations, respectively. As a sensitivity analysis, we repeated these models with additional adjustment for the frailty index and found little difference: estimates for A/H1N1, A/H3N2, and B following high-dose vaccine were 0.20 (0.08, 0.31), 0.13 (0.01, 0.26) and 0.27 (0.15, 0.40) standard deviations, respectively.

### CMV serostatus significantly moderates the association between biological age and vaccine responsiveness

**Fig. 3:**
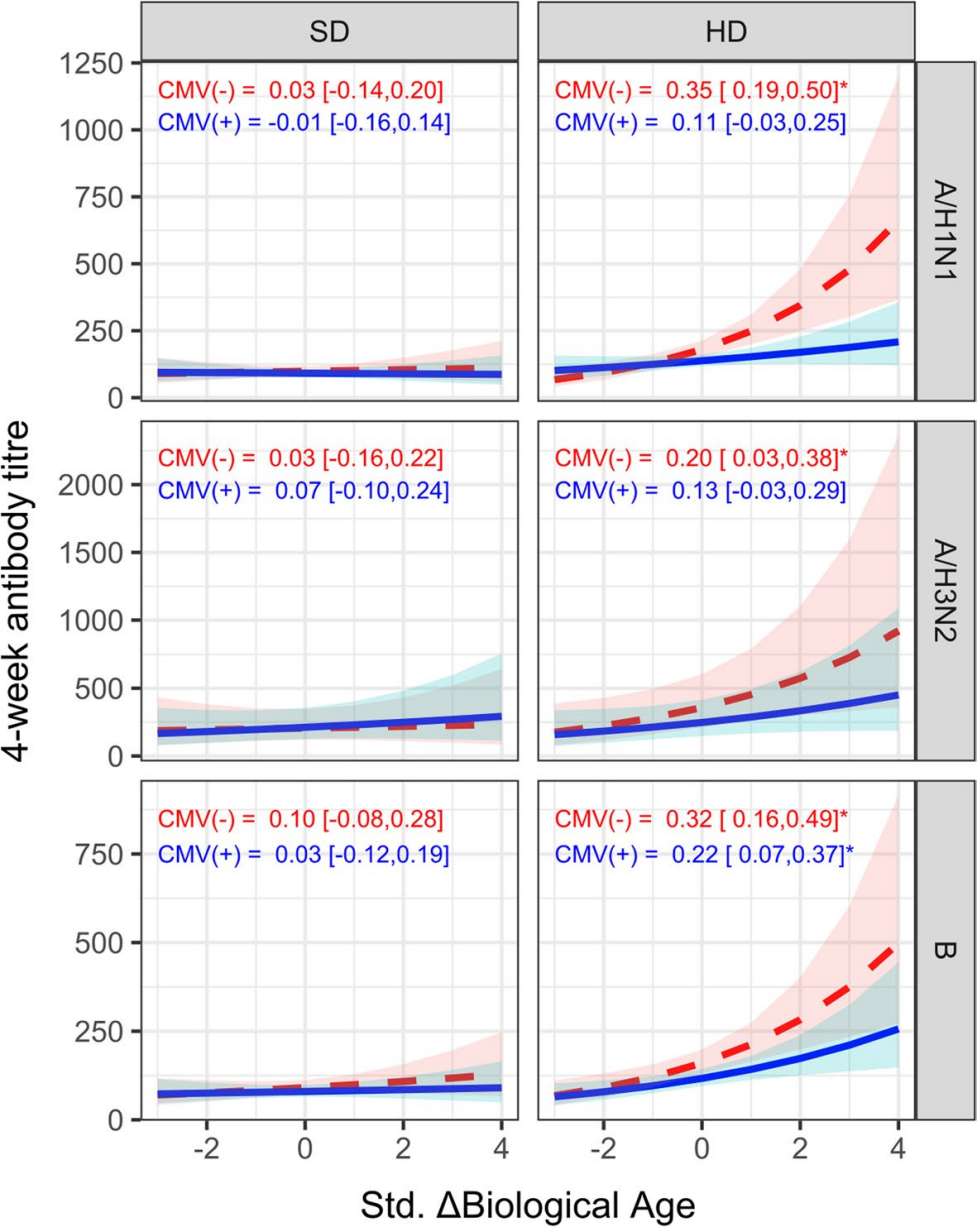
CMV serostatus modifies the association between biological aging (BA) and antibody responses in high dose recipients. The correlation between standardized ΔBA and 4-week antibody titre was estimated using mixed model regression, adjusting for the fixed effect of baseline titre and other covariates. Shown in plot is the estimated marginal mean of the exponentiated log 4-week titre and 95% confidence interval (CI) across the range of standardized BA for CMV negative (red, dashed line) and positive (blue, solid line) participants, stratified by vaccine dose. The standardized coefficient and 95% CI for the log 4-week titre is shown in each panel for CMV(-) and CMV(+) participants, and significance at *p* < 0.05 indicated with an asterisk

It has often been reported that CMV infection is correlated with weaker influenza vaccine responses [23] and therefore may moderate the association between biological age and vaccine immunogenicity. To test this hypothesis, we compared the estimated marginal means of antibody-titres 4-weeks post-vaccination over the range of biological age between CMV-positive and -negative participants (Fig. 3). For high-dose recipients, CMV positivity clearly abrogated the association between ΔBA and vaccine response, especially for A/H1N1 titres. For example, in CMV-negative and -positive participants, a 1-standard deviation change in ΔBA resulted in a 0.35 (95% CI: 0.19, 0.50) and 0.11 (- 0.03, 0.25) standard deviation increase in A/H1N1 titres, the interaction test for which was statistically significant in a subset analysis of high-dose recipients (ß = - 0.25, *p* = 0.041; Supplementary Table 1); this difference remained significant after adjusting for the frailty index (ß = - 0.28, *p* = 0.024; data not shown). CMV-negative participants also exhibited stronger A/H1N1 and B antibody titre responses than those who were CMV-positive (A/H3N2 = 0.20 (0.03, 0.38) vs. 0.13 (- 0.03, 0.29); B = 0.32 [0.16, 0.49] vs. 0.22 [0.07, 0.37]), although the interaction effect was not statistically significant for either (*p* > 0.40).

## Discussion

There is considerable heterogeneity in vaccine-induced protection against serious outcomes of influenza in older adults, which is typically studied in terms antibody-responses providing sterilizing immunity which are proven correlates of protection [25]. Although a number of immunological factors have been postulated as mediators of impaired vaccine-responses with age, the upstream biological mechanisms are poorly understood. We tested the hypothesis that variation in the extent of biological aging – the age-related loss of integrity and functionality of multiple physiological systems – would explain differences in vaccine immunogenicity in community-dwelling adults aged 65 and older. We quantified biological age using the previously validated KDM algorithm and a panel of 10 blood biomarkers reflecting integrity of several organ systems. In both the CLSA training sample and the vaccine trial sample, the measure of biological age was positively associated with chronological age (r ~ 0.6) and with frailty (Cohen’s d [26]~ 3–7.5). However, in contrast to our expectation, those with older biological age showed improved A/H1N1, A/H3N2 and B antibody titres post-vaccination, although this association was present only for high-dose participants and was diminished in those who were CMV seropositive. Even in this group, effect-sizes were small (d = 0.16–0.26). We also investigated the association between biological age and the incidence of break-through infections, but did not observe any trends that would support a relationship.

Given the lack of data pertaining to biological aging and immunity, it is difficult to explain why vaccine responses would increase with biological age. Aspects of T- and B-cell function, including activation, chemotaxis and proliferation [27, 28], and the frequency of naïve T- and B-cell populations [29] tend to decrease with age. However, IFN-? producing CD4^+^ T-cells, which promote strong vaccine responses through dendritic cell activation and enhanced antigen presentation [9], have been shown to be elevated in older adults [30–32]. This has been similarly shown for cell subsets that produce interleukin-10, a cytokine that promotes B-cell survival, proliferation and isotype switching [33], as well as germinal centre B-cell responses to vaccination [34]; specifically, IL-10 secreting T-regulatory cells increase with age [35, 36], as does monocyte IL-10 production following influenza vaccination in older adults [37]. It is also important to consider that these age-related changes may not be sufficient to strengthen antibody responses to standard-dose vaccine, hence why we only observed biological age to be associated with immunogenicity in high-dose recipients. This is supported by recent work showing that the relative effectiveness of high-dose over standard-dose vaccine is consistently higher in adults 85 and over compared to those 65 to 84 [38], as well as our own findings pertaining to frailty [39] and chronic inflammation (Picard et al., 2022: manuscript in press). Interestingly, and in line with aforementioned literature, the high-dose influenza vaccine is particularly effective at inducing the generation of antibody-producing plasmablasts [40], a process that is known to be enhanced by IFN-? producing CD4^+^ T-cells [41] and IL-10 [42].

Our observation of the association between biological age and vaccine responses being diminished in CMV seropositive individuals may also shed light on the underlying cellular mechanisms involved. Although the literature is inconsistent regarding the impact of CMV infection on influenza antibody responses [23], its influence on the T-cell compartment is well-documented. Following primary infection, which eventually occurs in up to 90% of adults worldwide, CMV enters a latent phase and over time T-cells that are specific to immunodominant epitopes on the virus can accumulate in great numbers; this is commonly referred to as memory inflation [43]. Specifically, CMV positivity is associated with an increased frequency of memory and terminally-differentiated CD8^+^ cells [43] and reduced frequency of influenza-specific IFN-? producing CD4^+^ cells [44, 45]. These effects, especially in the context of CD4^+^ cells, would likely counteract any beneficial synergism between high-dose vaccination and biological aging in improving vaccine antibody responses. Additional consequences of CMV infection, such as the reduction of switched memory B-cells [46], would also be expected to hamper possible benefits. Finally, in contrast to studies measuring biological age from DNA methylation [47, 48], we did not observe an association between biological age and CMV seropositivity. DNA methylation measures of biological aging are greatly influenced by changes in T- and NK-cell pools that also change with CMV infection [49], such as more frequent naïve and less frequent activated and memory subsets [43]. Hence, more studies are required to determine whether CMV actually plays a role in the multisystem physiological breakdown that represents biological aging.

Our study has notable strengths. It was nested within a previously published clinical trial conducted over 4 consecutive seasons where dose was randomized each year and a large number of participants were CMV-seropositive. Also, our biological age algorithm was based on a previously validated toolkit, and employed a biomarker panel with broad physiological context and a relatively large, separate training cohort of adults of similar demographics. Unfortunately, we are unable to draw conclusions on adults younger than 65, were limited in our ability to test associations between biological age and breakthrough infections due to a relatively low sample size, and did not pursue the cellular mechanism behind our primary findings. This is vital and future work should focus on the dynamics of important immune cell populations, particularly changes from pre- to post-vaccination, in relation to biological aging, high-dose vaccine and CMV-serostatus. The frequency and function of B-cells, CD4^+^ follicular helper cells, and monocytes would be excellent candidates.

## Conclusions

Our findings suggest that advanced biological age further improves the positive effect of high-dose influenza vaccine on antibody titres in older adults, which is suppressed in the presence of CMV positivity. This three-way interaction between biological age, vaccine dose and CMV serostatus implicates a common immunological mechanism, although further study is needed to confirm this, and identify which cells are actually involved.

## Methods

### Cohort description

The current study was a secondary analysis of data and biosamples from a double-blind randomized controlled trial to compare the immunogenicity of a trivalent high-dose versus quadrivalent standard-dose formulation of the split-virus influenza vaccine (Fluzone, Sanofi Pasteur) in older adults recruited from communities belonging to and surrounding Greater Sudbury, Ontario, Canada, and Hartford, Connecticut, USA (ClinicalTrials.gov: NCT02297542). The design and protocol have been previously published [39]. Briefly, over four consecutive influenza seasons (October 2014 – April 2015, October 2015 – April 2016, October 2016 – April 2017, and October 2017 – April 2018), adults aged 65 years and older were enrolled, vaccinated and provided blood for immune testing. Influenza surveillance included weekly contact with study subjects to assess flu-like symptoms or acute respiratory infection (ARI) [50]. Upon documentation of an ARI, nasopharyngeal swabs were collected (within 5 days of onset of symptoms) for polymerase chain reaction (PCR) detection of influenza virus. Influenza illness was documented by PCR detection of influenza virus following an ARI or evidence of seroconversion at 20-weeks post-vaccination.

In each year, older participants were randomized 1:1 to receive either standard- or high-dose vaccine at each study site. Blood samples were collected at the pre-vaccination and 4-, 10- and 20-week post-vaccination visits. Participants were allowed to re-enroll in subsequent seasons. Over the 4 years, there were 612 enrollments of 246 unique individuals. The study protocol was approved by the Institutional Review Board of the University of Connecticut Health Centre (UCHC) and the Health Sciences North Research Ethics Board and all study participants provided written informed consent to participate in the study.

### Study participants

Study participants were characterized according to demographics, chronic medical conditions, and functional impairments. BMI was calculated using weight and height measurements derived from a physician’s scale and analyzed in relation to a clinically meaningful difference of 2 kg/m^2^ [51]. CMV serostatus was determined in serum at baseline (pre-vaccination) using a CMV IgG ELISA kit (Genesis Diagnostics Inc., Cambridgeshire, UK) according to the manufacturer’s instructions. Frailty was defined using three common approaches: 1) a frailty index (FI) was calculated based on 40 items representing accumulated health deficits across multiple systems, which has been previously employed in studies of this trial [39, 52]. Using the frailty index (FI), participants were classified as robust (FI < 0.10), pre-frail (0.10 = FI < 0.21) or frail (FI = 0.21); 2) the Fried Frailty Phenotype, a summative score of five core components of physical frailty including exhaustion, weakness, unintentional weight loss, slowness and low physical activity [53], was calculated and used to categorize participants and robust (0 components present), pre-frail (1 or 2 present) or frail (3 or more present); and 3) the Clinical Frailty Scale (CFS), which uses subjective evaluation of specific domains of frailty including comorbidity, function, and cognition to generate a score ranging from 1 (very fit) to 9 (terminally ill) [54], was used to classify participants as low (CFS < 4), mild (4 < CFS < 6) or high (CFS = 6) frailty.

### Hemagglutination-inhibition antibody titres

Hemagglutination-inhibition (HAI) antibody titres were quantified using previously-described standard methods [55]. Influenza types used for HAI testing were as follows: Year 1, A/Texas/50/2012, A/California/7/2009 and B/Massachusetts/2/2012; Year 2, A/Switzerland/9715292–2013, A/California/7/2009 and B/Phuket/3073/2013; Year 3, A/Hong Kong/4801–2014, A/California/7/2009 NYMC X-179A and B/Brisbane/60/2008; and Year 4, A/HongKong/4801/2014, A/Michigan/45/2015 and B/Brisbane/60/2008. Laboratory testing was conducted after each study year, and participant serum was randomized before plating.

### Estimation of biological age

A subset of 300 participant enrollments (representing 166 unique individuals over to 4-year trial) were randomly selected for the quantification of clinical chemistry and inflammatory blood biomarkers, described below. This maximized allotted laboratory resources and allowed for 80% power to detect very small to small effect sizes (d = 0.16, a = 0.05) [56] for our primary analysis of the association of vaccine antibody titres with biological age. Each biomarker was measured at the pre-vaccination baseline for every year that a given participant was enrolled.

We quantified biological age using the Klemera-Doubal method (KDM) [57] implemented within the ‘BioAge’ R package [58]. The KDM biological age is derived from integrating parameters estimated across a series of univariate regression models relating each of a panel of biomarkers to chronological age. The resulting biological age value can be interpreted as the chronological age at which an individual’s biomarker levels would appear typical in the reference population. We derived the KDM biological age algorithm for the vaccine trial from analysis of data from the Canadian Longitudinal Study of Aging (CLSA) [59]; this represents our training sample, from which parameters for the KDM algorithm are estimated. Briefly, we fitted regressions of each biomarker on chronological age in a sample of *n* = 3866 women and men aged 65 to 85 who participated in the baseline CLSA data collection (Baseline Comprehensive Dataset version 5.1). Regressions were fitted separately for women and men. Parameters from these regressions were then combined to form separate KDM biological age algorithms for women and men. KDM biological age parameters are reported in Supplementary Table 2.

We composed the KDM algorithm from ten clinical chemistry and inflammatory blood biomarkers purposely selected for this study based on our previous experience quantifying biological age [60] and their availability within the CLSA. The biomarkers were serum albumin (ALB), alanine aminotransferase (ALT), creatinine (CREAT), ferritin (FERR), free thyroxine (T4), cholesterol (CHOL), high-density lipoprotein (HDL), triglycerides (TRIG), TNF and IL-6. Biomarker measurements in the CLSA have been described previously [60]. In the CLSA training data, biological age was correlated with chronological age and was nearly centred on zero (Supplementary Fig. 1A,B), and those participants classified as frail or pre-frail in an independent analysis [61–63] tended to have older biological ages as compared to those classified as robust (ß [95% CI]: frail vs. robust = 3.23 years [2.69, 3.76]; pre-frail vs. robust = 1.16 years [0.77, 1.55]; Supplementary Fig. 1C).

In the vaccine trial, biomarkers were measured from blood samples collected at pre-vaccination baseline. TNF and IL-6 were measured from plasma using the Ella Automated Immunoassay System and Simple Plex 2nd generation Human TNF and IL-6 cartridges (R&D Systems, MN, USA). To adjust for differences in TNF and IL-6 due to measurement from plasma instead of serum, levels of each measure were adjusted prior to algorithm parametrization so that the sex-specific distribution was similar between cohorts; where *m* is the mean, *s* is the standard deviation and *x* is the biomarker level, *x*_*new*_ = *m*_*clsa*_ + (*x*_*old*_ - *m*_*vax*_) * (*s*_*clsa*_/*s*_*vax*_). All other biomarkers were measured from serum using standard protocols at the Health Sciences North clinical laboratory (Sudbury, ON).

We applied the KDM biological age algorithm developed in the CLSA data to compute biological age values for vaccine trial participants. We computed ΔBA as the difference between participant’s biological age and their chronological age. ΔBA values > 0 indicate more advanced biological aging and corresponding higher risk for disease, disability, and mortality. ΔBA values < 0 indicate the opposite. We also stratified participants into younger, average and older groups according to sex-specific tertiles in BA.

### Statistical analysis

Continuous data were summarized as the mean or geometric mean and standard deviation and categorical as the count and frequency. Where applicable, crude bivariate comparisons were performed by Student’s T-test, Fisher’s exact test or Pearson’s correlation test.

Regression modelling was performed using a mixed model approach using the ‘lme4’ package in R. For models where natural-log antibody titre was the dependant variable, age, sex, and a two-way ΔBA*dose or three-way ΔBA*dose*CMV interaction were included as fixed effects, along with random intercepts for participant and year; to model the change in antibody response over time, the natural-log baseline titre was also included as a fixed effect. Where standardized coefficients are presented, the natural-log titre and BA were transformed to have a mean of 0 and standard deviation of 1 to facilitate comparability across virus subtypes; hence, coefficients represent the proportional standard deviation change in antibody titre per 1-standard deviation change in ΔBA. In models that included interactions, coefficients for ΔBA within strata were calculated using the ‘emmeans’ package in R, and to plot estimated marginal means for antibody titres across the range of ΔBA, the ‘sjPlot’ package in R was used.

All analyses were performed in the R environment, version 4.0.2.

## Supplementary Information

**Additional file 1: Supplementary Table 1.** Regression table describing the association between standardized, natural-log 4-week antibody titres and ΔBA in high-dose recipients, including a two-way interaction between ΔBA and CMV serostatus. **Supplementary Table 2.** KDM training parameters derived from the CLSA and used calculate biological aging in the vaccination cohort. **Supplementary Figure 1.** Characteristics of KDM biological age (BA) in the CLSA training cohort and associations with the frailty index (FI). A) Pearson’s correlation (R) for BA with chronological age and significance (p) for females (red asterisk) and males (blue dots). B) Distribution of ΔBA in females and males, including the mean and standard deviation. C) Differences in years for ΔBA (and 95% confidence interval) for pre-frail (0.10=FI<0.21) and frail (FI=0.21) individuals relative to robust (FI<0.10) individuals; estimates calculated using univariate regression.

## Data Availability

The datasets used and/or analysed during the current study are available from the corresponding author on reasonable request.
