# Supplementary material for: Advanced biological age is associated with improved antibody responses in older high-dose influenza vaccine recipients over four consecutive seasons
Source: Immun Ageing. 2022 Aug 23;19:39. doi: 10.1186/s12979-022-00296-7 (PMC9396565; doi:10.1186/s12979-022-00296-7)

# Supplementary materials

**Supplementary Table 1:** Regression table describing the association between standardized, natural-log 4-week antibody titres and ΔBA in high-dose recipients, including a two-way interaction between ΔBA and CMV serostatus.

|  | **Std. A/H1N1 [log]** | | | **Std. A/H3N2 [log]** | | | **Std. B [log]** | | |
| --- | --- | --- | --- | --- | --- | --- | --- | --- | --- |
| *Predictors* | *Estimates* | *CI* | *p* | *Estimates* | *CI* | *p* | *Estimates* | *CI* | *p* |
| (Intercept) | -2.44 | -3.96 – -0.92 | **0.002** | -2.68 | -4.24 – -1.12 | **0.001** | -2.05 | -3.37 – -0.74 | **0.002** |
| Baseline titre [log] | 0.79 | 0.64 – 0.94 | **<0.001** | 0.57 | 0.44 – 0.71 | **<0.001** | 0.8 | 0.64 – 0.97 | **<0.001** |
| Age | 0 | -0.02 – 0.02 | 0.979 | 0.01 | -0.01 – 0.03 | 0.327 | 0 | -0.02 – 0.01 | 0.627 |
| Sex [Male] | -0.26 | -0.54 – 0.02 | 0.064 | -0.09 | -0.37 – 0.20 | 0.548 | -0.29 | -0.55 – -0.04 | **0.023** |
| ΔBA | 0.35 | 0.17 – 0.54 | **<0.001** | 0.19 | -0.00 – 0.38 | 0.054 | 0.31 | 0.13 – 0.50 | **0.001** |
| CMV [Positive] | -0.26 | -0.53 – 0.01 | 0.06 | -0.31 | -0.59 – -0.03 | **0.031** | -0.33 | -0.59 – -0.08 | **0.01** |
| ΔBA * CMV [Positive] | -0.25 | -0.49 – -0.01 | **0.041** | -0.05 | -0.30 – 0.20 | 0.706 | -0.1 | -0.34 – 0.14 | 0.409 |
| **Random Effects** | | | |  | | |  | | |
| σ^2^ | 0.26 | | | 0.3 | | | 0.44 | | |
| τ_00_ _ComboID_ | 0.27 | | | 0.27 | | | 0.06 | | |
| τ_00_ _Year_ | 0.01 | | | 0.2 | | | 0.05 | | |
| ICC | 0.51 | | | 0.62 | | | 0.2 | | |
| N _ComboID_ | 110 | | | 110 | | | 110 | | |
| N _Year_ | 4 | | | 4 | | | 4 | | |
| Observations | 147 | | | 147 | | | 147 | | |
| Marginal R^2^ / Conditional R^2^ | 0.549 / 0.781 | | | 0.321 / 0.739 | | | 0.503 / 0.603 | | |

**Supplementary Table 2:** KDM training parameters derived from the CLSA and used calculate biological aging in the vaccination cohort.

| Biomarker | Sex | N | q | k | RMSE | r2 |
| --- | --- | --- | --- | --- | --- | --- |
| ALB | Female | 1928 | 3.7741 | -0.001 | 0.068 | 0.0144 |
|  | Male | 1938 | 3.8019 | -0.002 | 0.068 | 0.0205 |
| ALT | Female | 1928 | 3.6409 | -0.01 | 0.378 | 0.0212 |
|  | Male | 1938 | 4.1681 | -0.015 | 0.386 | 0.0457 |
| CHOL | Female | 1928 | 1.8995 | -0.003 | 0.21 | 0.0081 |
|  | Male | 1938 | 1.8659 | -0.005 | 0.236 | 0.0148 |
| CREAT | Female | 1928 | 3.9243 | 0.005 | 0.199 | 0.0204 |
|  | Male | 1938 | 4.0431 | 0.0066 | 0.21 | 0.0309 |
| FERR | Female | 1928 | 4.7502 | -0.002 | 0.758 | 0.0001 |
|  | Male | 1938 | 5.3775 | -0.005 | 0.778 | 0.0014 |
| HDL | Female | 1928 | 0.424 | 0.0007 | 0.285 | 0.0002 |
|  | Male | 1938 | 0.0695 | 0.0025 | 0.289 | 0.0024 |
| IL6 | Female | 1928 | -0.381 | 0.0166 | 0.572 | 0.0265 |
|  | Male | 1938 | -0.932 | 0.0243 | 0.554 | 0.0581 |
| T4 | Female | 1928 | 2.5883 | 0.002 | 0.178 | 0.0040 |
|  | Male | 1938 | 2.6435 | 0.0008 | 0.151 | 0.0008 |
| TNF | Female | 1928 | -0.923 | 0.0142 | 0.355 | 0.0494 |
|  | Male | 1938 | -0.857 | 0.0136 | 0.33 | 0.0522 |
| TRIG | Female | 1928 | 0.6191 | -0.003 | 0.462 | 0.0010 |
|  | Male | 1938 | 1.1232 | -0.01 | 0.496 | 0.0115 |

Parameters derived from univariate regressions of each biomarker on chronological age: N, sample size; q, intercept; k, beta coefficient; RMSE, root mean square error; r2, r-squared.

**Supplementary Figure 1:** Characteristics of KDM biological age (BA) in the CLSA training cohort and associations with the frailty index (FI). A) Pearson’s correlation (R) for BA with chronological age and significance (p) for females (red asterisk) and males (blue dots). B) Distribution of ΔBA in females and males, including the mean and standard deviation. C) Differences in years for ΔBA (and 95% confidence interval) for pre-frail (0.10≤FI<0.21) and frail (FI≥0.21) individuals relative to robust (FI<0.10) individuals; estimates calculated using univariate regression.


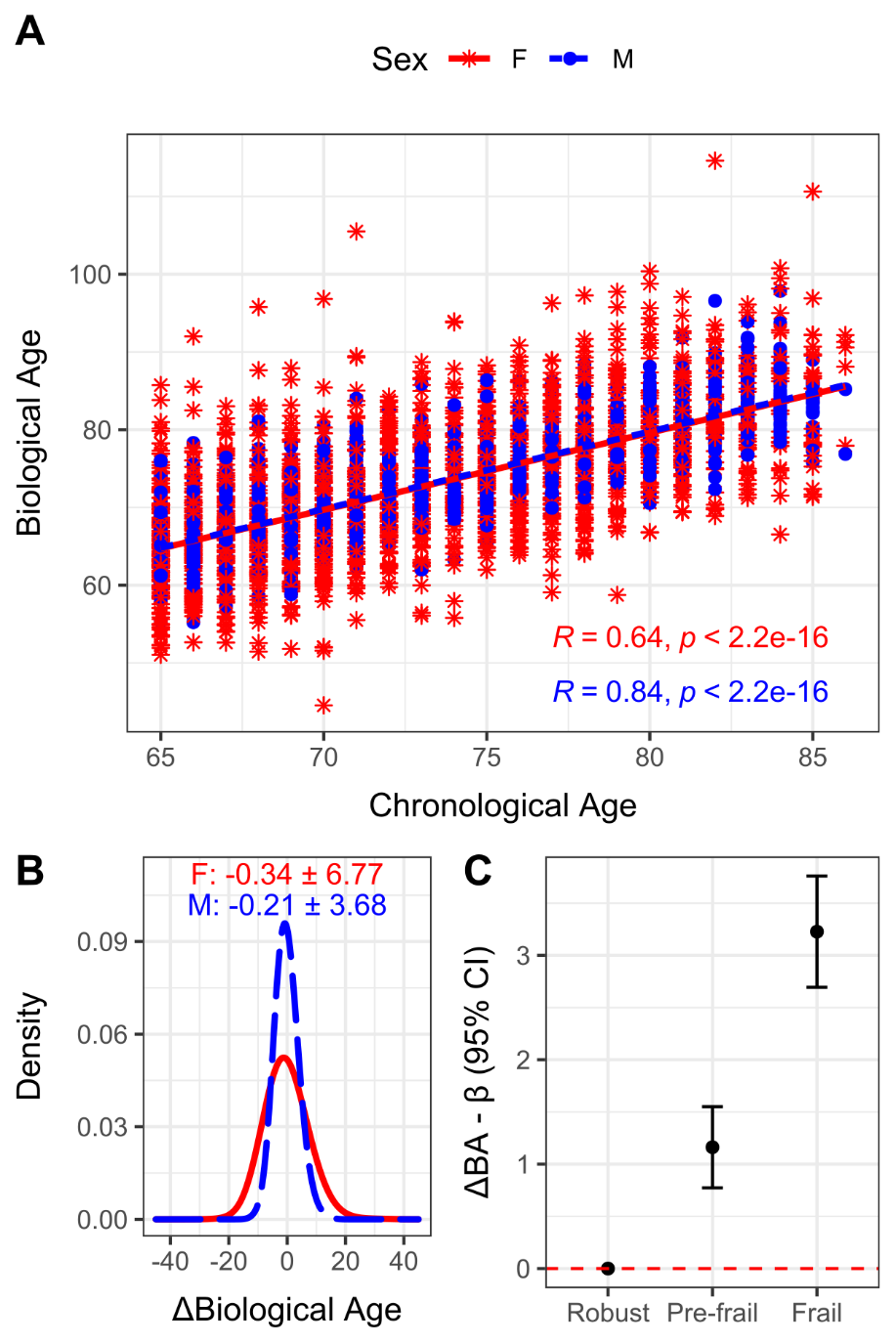

Supplement: Supplementary file 1 — Additional file 1: Supplementary Table 1. Regression table describing the association between standardized, natural-log 4-week antibody titres and ΔBA in high-dose recipients, including a two-way interaction between ΔBA and CMV serostatus. Supplementary Table 2. KDM training parameters derived from the CLSA and used calculate biological aging in the vaccination cohort. Supplementary Figure 1. Characteristics of KDM biological age (BA) in the CLSA training cohort and associations with the frailty index (FI). A) Pearson’s correlation (R) for BA with chronological age and significance (p) for females (red asterisk) and males (blue dots). B) Distribution of ΔBA in females and males, including the mean and standard deviation. C) Differences in years for ΔBA (and 95% confidence interval) for pre-frail (0.10=FI<0.21) and frail (FI=0.21) individuals relative to robust (FI<0.10) individuals; estimates calculated using univariate regression. [file 12979_2022_296_MOESM1_ESM.docx]
